# Left to Their Own Devices: Breakdowns in United States Medical Device Premarket Review

**DOI:** 10.1371/journal.pmed.1000280

**Published:** 2010-07-13

**Authors:** Jonas Zajac Hines, Peter Lurie, Eunice Yu, Sidney Wolfe

**Affiliations:** Health Research Group at Public Citizen, Washington, D.C., United States of America

## Abstract

Using examples from recent FDA regulatory proceedings, Jonas Hines and colleagues critique the medical device premarket review and identify eight weaknesses in the process that should be remedied.

**Summary Points**

- The number and complexity of medical devices have increased over the past several decades. A series of recent safety issues have raised public awareness about shortcomings in the Food and Drug Administration's (FDA) regulation of medical devices.
- We provide a background on medical device premarket review and identify eight addressable weaknesses in the process.
- These include a lower approval standard than their drug counterparts, excessive reliance upon a fast-track process, and failure to conduct Congressionally mandated device classifcations.
- Paradigmatic cases drawn from recent Food and Drug Administration regulatory proceedings illustrate each weakness.

## Introduction

Medical devices encompass nearly every medical product that does not achieve its intended purpose through chemical action, from the simple (tongue blades) to the complex (MRI machines), and from the safe (stethoscopes) to the risky (artificial hearts) [1],[2]. Certain drug–device combinations, such as drug-eluting coronary stents, are also regulated as devices.

The number and complexity of medical devices have increased dramatically over the past several decades, often to the betterment of patients' health. Between 1997 and 2006, the value of device sales roughly doubled to US$123 billion, representing a fairly consistent 6% of the nation's health care expenditures [3].

The Center for Devices and Radiological Health (CDRH) at the Food and Drug Administration (FDA) is charged with ensuring the safety and effectiveness of medical devices. While a number of serious safety problems with devices have emerged—the Dalkon Shield [4], the Bjork-Shiley heart valve [5], and the Sprint Fidelis defibrillator lead [6], to name a few—problems with effectiveness are not as readily apparent once a device is on the market, in part because postmarket efficacy trials of approved devices are rare. Thus, the burden of ensuring device effectiveness is heavily weighted toward premarket evaluation.

In this article, we first review the history of premarket device regulation at CDRH and then identify eight addressable weaknesses at the FDA level and above that impede the agency's ability to review devices for efficacy, each accompanied by paradigmatic cases from recent regulatory proceedings. Table 1 summarizes these weaknesses according to the type of remedial action required. The cases are intended only to be illustrative and do not represent a random subset of FDA device approvals. Because of FDA policies prohibiting the release of data on unapproved products, we are unable to estimate the prevalence of these problems. Moreover, we do not evaluate each of the approximately 3,000 applications approved or cleared by CDRH each year. Other aspects of medical device regulation, such as postmarketing surveillance, modifications to already-approved devices, and manufacturing facility inspection, are beyond the scope of this article.

**Table 1:** Summary of statutory and regulatory issues, case exemplars, and necessary corrective actions.

		Case Exemplar	Definitive Action Needed	Immediate Shifts in Agency Discretionary Practices
*Problems requiring statutory actions* a				
Issue 1	Lower approval standard for devices than for drugs	Vagus nerve stimulator	Amend 21 USC § 360c to require treatment devices to meet the same standard as drugs	Insist on higher standards
Issue 3	Disparate technological characteristics	Transcranial magnetic stimulation	Repeal 21 USC § 360c(i)(1)(A)(ii) to prohibit such comparisons	Conservative application in limited number of cases
Issue 4	*De novo* process	Transcranial magnetic stimulation	Repeal 21 USC § 360c(f)(2)	Limited use for only devices which are low risk
Issue 8	Unique appeal mechanism for device manufacturers	Intergel adhesion barrier	Repeal 21 USC § 360e(g)(2)	Use other established dispute resolution routes that already exist for pharmaceuticals and biologics
*Problems requiring regulatory actions* a				
Issue 2	Permissive interpretation of “same intended use”	Collagen scaffold	Regulation defining criteria for determining “same intended use”	Tighten agency interpretation of “same intended use”
Issue 5	Predicate creep	Pathwork tissue of origin test	See actions for issues 2 and 3	See shifts in agency practice for issues 2 and 3
*Problems requiring changes in discretionary practices*				
Issue 6	Failure to complete review of class III 510(k) devices	Intraaortic balloon pump	Complete classification of such devices, requiring PMA applications for those retained in class III	Same as definitive action
Issue 7	Some devices have never been classified	Heart valve allograft	Complete classification of all unclassified preamendments devices	Same as definitive action

a Although these weaknesses are susceptible to shifts in agency discretionary practices, such changes are not sufficient; for consistent and meaningful improvement, these laws and regulations must be revisited and strengthened.

## History and Background of Premarket Device Review

**Figure 1:**
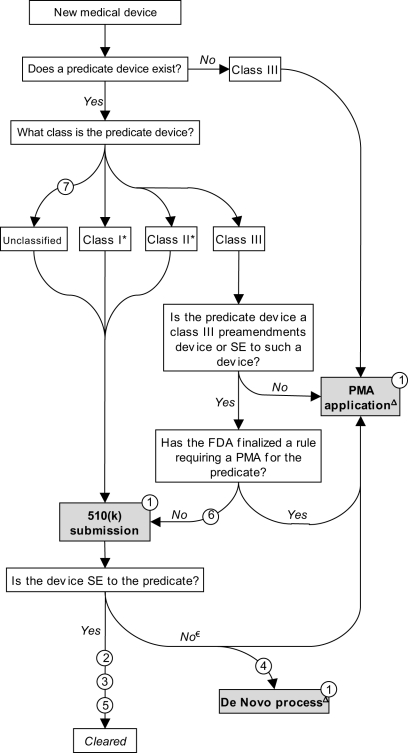
Schematic representation of medical device premarket review mechanisms. *Note*: Issues listed in circles. Issue 8 does not appear in Figure 1. SE, substantially equivalent. * The 1997 FDAMA exempted most class I devices and a small number of class II devices from 510(k) requirements. € If determined to be not substantially equivalent, the sponsor may submit a PMA application. Alternatively, a sponsor may request evaluation under the *de novo* pathway (see text). Δ Post-decision scheme not illustrated.

For much of the twentieth century, medical devices were largely unregulated and the vast majority were not subject to any premarket review. To address this vacuum, in 1976 Congress passed the landmark Medical Device Amendments (MDA) to the federal Food, Drug, and Cosmetic Act (FDCA). Its primary purpose was to prevent the distribution of dangerous and ineffective devices by creating a comprehensive premarket review mechanism. This was accomplished by steering new devices through one of two premarket review procedures — “premarket approval” (PMA) and “premarket notification,” the latter often referred to as “510(k)” after the relevant section of the FDCA — determined by a three-tiered scheme that stratifies devices into “classes” corresponding to their potential risks (Figure 1) [7],[8].

A PMA application is analogous to a New Drug Application (NDA). Sponsors must submit valid scientific evidence, generally based on clinical trials, that directly establishes safety and efficacy. By contrast, in a 510(k) submission, a sponsor establishes that a device is safe and effective by demonstrating only that the new device is “substantially equivalent” to an existing (“predicate”) 510(k) device [9]. Substantial equivalence is evaluated according to the *intended use* of the product and its *technological characteristics* [10]. Once a device is cleared as a 510(k), it may serve as a predicate device for subsequent 510(k) submissions.

Class III devices are high-risk, or novel, devices and most require direct demonstration of safety and effectiveness through the PMA pathway. Class II devices present moderate risks to patients; in most cases, manufacturers must submit 510(k)s before marketing. Class I devices are low-risk and most are currently exempted from any premarket review [11]; they are subject only to rudimentary controls such as product listing and labeling. From fiscal years 2003–2007, roughly 50,000 devices entered the market, of which 2%, 26%, and 71% were class III, II, and I, respectively (these figures include applications for postmarket modifications, an issue not covered in this article). During this period, 79% of class III devices went through PMA, with the remaining proceeding through 510(k) (*see Issue 6 below*). Fourteen and 96% of class II and I devices were 510(k)-exempt, respectively [11].

## Lower Approval Standard for Medical Devices Than for Drugs (Issue 1)

Regardless of whether the product is reviewed under PMA or 510(k), by statute the approval standard for medical devices is lower than for drugs. Before a new drug can be marketed, the sponsor must show “substantial evidence [of effectiveness],” [12] whereas the sponsor of a new device need only demonstrate a “reasonable assurance of … safety and effectiveness” [13]. In practice, NDAs typically contain two or more well-controlled clinical studies [14], whereas for PMA applications, a single study is the norm [15] and most 510(k)s contain no clinical data [16]. While for drugs, “uncontrolled studies or partially controlled studies are not acceptable as the sole basis for the approval of claims of effectiveness,” [17] for devices, the regulations permit “reliance upon other valid scientific evidence … even in the absence of well-controlled investigations” [18]. Thus, data that would never be sufficient to support the approval of a drug can result in the approval of a device used to treat the same condition, potentially diverting patients from effective drugs to less-effective devices.

This concern is not merely theoretical. Consider the vagus nerve stimulator (VNS), a surgically implanted device for treatment-resistant depression. In the only randomized controlled trial (RCT), the device did not demonstrate a statistically significant benefit on the primary measure of depression at ten weeks (p = 0.25) [19]. However, in its PMA application, the company relied on follow-up data at one year in which treated patients were claimed to have improved more than a non-randomized, unblinded, non-concurrent control group (p<0.001); both groups were also permitted co-interventions. A psychopharmacology expert in the FDA's drug center advised CDRH that, with similar data for an antidepressant drug, the center would not have permitted the filing of an NDA, adding, “it is artificial to us to consider one study for a device (that is negative on face) as sufficient to provide evidence for regulatory efficacy when we require positive studies for a drug” [20]. While CDRH initially issued a non-approvable letter, the director of CDRH reversed this decision and approved the device, overruling more than 20 FDA scientists and officials [20].

Subsequently, the Centers for Medicare and Medicaid Services determined that VNS was not “reasonable and necessary,” the standard for reimbursement under Medicare. Moreover, it did “not believe there is a treatment benefit directly attributable to VNS” [21]. Other third-party payers have also denied coverage for this expensive device [22].

## Reliance upon Less-Rigorous Review Mechanisms

Compared to the PMA process, the 510(k) review is “generally less stringent … less expensive … [and] faster” [11]. The average total review time for review of 510(k) submissions in fiscal year 2006 was 54 days, whereas for PMA applications it was 283 days [23]. Unlike PMA applications, direct evidence of safety and effectiveness is usually not required for 510(k) submissions [9]; only 10–15% of 510(k) submissions contain any clinical data [16]. Instead, 510(k) submissions primarily contain performance characteristics comparing a new device to a predicate. In considering a PMA application, the FDA may consult with an advisory committee comprised of non-government experts; this option is rarely pursued for 510(k) submissions. As the FDA acknowledges, it “does not attempt to address all of the issues [that] would be answered in a PMA in its review of 510(k)s” [9]. Finally, whereas the FDA has explicit authority to recall or temporarily suspend marketing of PMA-approved devices [24], corresponding statutory language does not exist for 510(k)-cleared devices.

### Permissive Interpretation of “Same Intended Use” (Issue 2)

According to FDA practices subsequently codified in the Safe Medical Devices Act of 1990 (SMDA), a device must have the same intended use as its predicate for clearance under the 510(k) process. However, the intended use of a product and its labeled indication are not synonymous [25]. In the absence of a statutory definition of “same intended use,” agency practice permits a lenient interpretation of this term; the agency asserts that its “scientific expertise enables it to exercise considerable discretion in construing intended uses” [9]. In practice, the FDA has permitted even novel implantable devices to be reviewed under the 510(k) process.

For example, ReGen's Menaflex Collagen Scaffold (MCS) is a device implanted during arthroscopic surgery to replace a damaged medial meniscus. After consulting with the FDA, which determined that the MCS belonged in class III [26], ReGen began a trial to support a PMA application [27]—a two-year RCT comparing partial meniscectomy to partial meniscectomy with MCS implantation—with the final patient evaluated in May 2005 [28]. The trial failed to show any benefit for the MCS on all three primary clinical endpoints [29],[30]. In December 2005, the FDA allowed the company to shift courses and submit a 510(k) claiming that the MCS was a surgical mesh. This, and another 510(k) submitted in December 2006, were both rejected by the agency.

In a third attempt submitted in July 2008, ReGen again claimed the MCS was substantially equivalent to surgical meshes (e.g., rotator cuff mesh, anal fistula plug, hernia repair graft, pelvic floor reconstruction mesh). However, as an FDA reviewer pointed out [30], none of these meshes are implanted in a weight-bearing joint or intended to facilitate the regrowth of articular cartilage.

The company downplayed the results of the RCT and argued that it was entitled to the less-rigorous review given to the MCS's predicate devices. It claimed that bench testing data (e.g., suture retention strength and tensile strength) should provide the primary basis for establishing substantial equivalence [31]. Articulating this point before an FDA advisory committee, the company asserted that the committee's decision should be based upon “the function of this device as a surgical mesh … and not the ultimate clinical outcome” [32]. After a favorable advisory committee review, the FDA cleared the MCS for commercial distribution in December 2008.

In September 2009, the FDA released a preliminary report criticizing its own handling of the MCS's premarket review [27]. The report described a contentious review process, with the FDA ultimately acceding to intense pressure from ReGen and its Congressional advocates by altering its typical review procedures. Irregularities included unusual involvement of senior FDA leadership—including the then-FDA commissioner—in decisions usually made at lower levels, a shortened review time, and replacement of several standing advisory committee members with clinicians in specialties thought more likely to favor the device. In addition, the report described the replacement of the FDA review team by an FDA official (thought by the company to be more likely to be favorably disposed toward the device) to present the agency's findings before the advisory committee. Lastly, the report described over reliance on the advisory committee's recommendation in clearing the MCS and highlighted disagreement within the agency over the interpretation of “same intended use” employed in this case. The FDA is currently reevaluating its clearance of the MCS. The Centers for Medicare and Medicaid Services has proposed denying reimbursement for the MCS on the grounds that “the evidence is adequate to conclude that the collagen meniscus implant does not improve health outcomes” [33].

### Disparate Technological Characteristics (Issue 3)

The other criterion for substantial equivalence, also codified in the SMDA, relates to the technological characteristics of a new device and its predicate. Differences in such characteristics do not preclude a finding of substantial equivalence, as long as the differences do not raise new issues of safety or effectiveness [34]. Indeed, 14% of cleared 510(k) submissions have different technological characteristics than their predicates [11]. This provision has led to devices acting as predicates for markedly dissimilar devices.

For instance, the transcranial magnetic stimulation (TMS) device is intended to treat depression by applying a magnetic field to a specific region of the brain. The agency permitted TMS to be reviewed under the 510(k) process with electroconvulsive therapy (ECT) as the predicate device, even though ECT involves the administration of electrical currents to induce a generalized seizure. Despite this claim of equivalence, the manufacturer, Neuronetics, provided no information suggesting it conducted any studies directly comparing the two devices [35]; instead, it conducted a nine-week RCT comparing TMS to a placebo. The difference between patients treated with active and sham TMS was clinically minor (1.7 points on a 60-point scale) and statistically non-significant (*p* = 0.057); only the post hoc exclusion of six patients who had met a priori inclusion criteria yielded statistical significance (*p* = 0.038) [36],[37]. An advisory committee concluded that “the clinical effect was perhaps marginal, borderline, questionable, and perhaps a reasonable person could ask whether there was an effect at all” [38]. The FDA subsequently determined that TMS was not substantially equivalent to ECT.

### De Novo Process (Issue 4)

However, Neuronetics persisted and TMS ultimately reached the market via a relatively obscure premarket review procedure called the de novo process. Created in the FDA Modernization Act of 1997 (FDAMA) as a means to permit low-risk, novel devices onto the market without a PMA, it is reserved only for devices previously denied clearance in the 510(k) pathway. Under this pathway, the sponsor of a rejected product may request clearance without identifying a predicate device, thus circumventing another 510(k) or even a PMA [39]. Here, the company requested clearance for a modified indication identified by a questionable post hoc analysis [40],[41] of the negative RCT. Importantly, Neuronetics could not have used the de novo process without the initial 510(k) designation, which itself was made possible only by permitting technologically dissimilar devices to use the 510(k) pathway. Since the de novo process was created, 52 devices have been cleared through this pathway [42].

### Predicate Creep (Issue 5)

The 510(k) process allows sponsors to identify a predicate device that was itself substantially equivalent to another device that was substantially equivalent to another, and so on. This iterative process permits a scenario in which, over multiple cycles, a new device can be quite dissimilar to the original predicate device—so-called “predicate creep” [11],[27].

For example, the Pathwork Tissue of Origin Test, cleared in 2008, is a microarray kit that compares the RNA expression pattern from a tumor with an unknown primary to the expression patterns of 15 common tumors [43]. This device's predicate device was the BioPlex 2200 Medical Decision Support Software, a software algorithm cleared in 2005 that assists in diagnosing autoimmune disorders by matching enzyme-linked immunoassay results to a database of sera from patients with autoimmune disorders [44]. This device, in turn, had been declared substantially equivalent to the Remedi HS Drug Profiling System, an algorithm-based diagnostic kit cleared in 1995 that tests for illicit drugs. Thus, a screening test for illicit drugs ultimately allowed for the clearance of a malignancy diagnostic test, simply because both use computer programs to compare samples to an existing database.

## Failure to Implement Statutory Requirements

When the MDA was enacted, more than 1,700 types of devices were already in commercial distribution [45], the so-called “preamendments” devices. Although their continued marketing was permitted, the FDA was required to assemble expert panels to assign them to one of the three medical device classes, which the FDA then finalized. The FDA finalized the last panel recommendations in 1988 [46].

As a result, any class I or II preamendments device could remain on the market without submitting a 510(k) [47]. Class III preamendments devices (approximately 8% of preamendments device types [45]) were permitted to remain on the market until the FDA finalized a rule calling for a PMA application for that type of device; until then, these devices could serve as predicates in subsequent 510(k) submissions for class III devices [8].

### Failure to Complete Review of Class III 510(k) Devices (Issue 6)

With scarce resources and new devices continually entering the market, the FDA was slow to call for PMA applications for these devices. In the 1990 SMDA, Congress expanded the definition of class II to include some devices previously considered class III. Thus, Congress required the agency to revisit the class III preamendments device types still regulated under 510(k) to either reclassify them or issue a rule requiring a PMA application by December 1, 1996 [48],[49].

**Figure 2:**
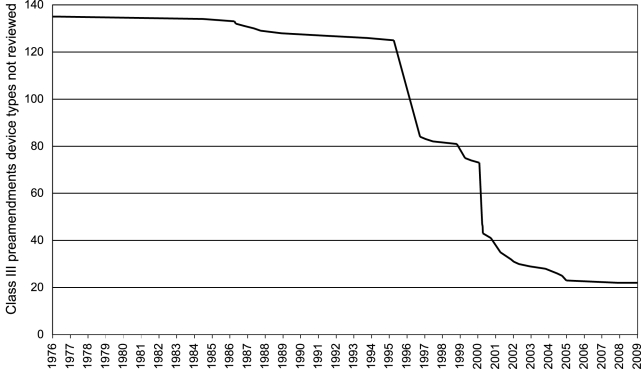
Class III 510(k) device types not fully reviewed by the FDA, 1976–2009. *Note*: if a device was later determined to have more than one indication, the review was considered complete only after all indications had been reviewed.

We tracked the 135 class III preamendments device types identified by the FDA in 1994 to establish their current regulatory status (Figure 2) [48]. The FDA had issued regulations for only 5% (seven types) of these devices at the time of the 1990 SMDA. By the 1996 deadline, it had complied with the statutory mandate for only 38% of class III preamendments devices. At present, the FDA still has not completed regulatory proceedings for 22 of the original 135 class III preamendments device types (16%), allowing them to continue to serve as predicate devices under the 510(k) process. According to a recent United States Government Accountability Office report [11], two-thirds of all class III preamendments devices cleared from FY2003–2007 were implanted, life-sustaining, or posed a significant risk. Responding to this report, the FDA recently initiated the process of determining whether a PMA would be required for most of the remaining devices [50].

ECT, certain pacemakers and pacemaker leads, hemodialysis shunts, and certain cardiopulmonary bypass pumps are all class III devices still cleared through the 510(k) pathway. This enabled Neuronetics to file a 510(k) submission, rather than a PMA, for TMS. Intra-aortic balloon pumps are also class III preamendments devices currently cleared through the 510(k) pathway, most without the provision of clinical data [51]. The devices have been associated with rare but serious complications, including severe bleeding, limb ischemia, and death [52].

### Some Devices Have Never Been Classified (Issue 7)

More than 200 types of preamendments devices have never been placed into class I, II, or III [53]. Overlooked by the FDA in the original classification process, these devices proceed through the 510(k) pathway using other unclassified devices as predicates. Examples include silicone pectoralis muscle implants, malar implants, and certain vertebral body internal fixation devices.

### Manufacturer Appeal Mechanisms Unique to Center for Devices and Radiologic Health (Issue 8)

In 1997, the FDAMA created a Medical Devices Dispute Resolution Panel (MDDRP), an external panel intended to help resolve scientific disputes between sponsors and the FDA [54]. This panel, unique to CDRH, provides a sponsor with another opportunity to secure a favorable outcome, even after the FDA has formally rejected its device. It has been used three times. The first product to come before the MDDRP was Lifecore's Intergel, a solution instilled into the peritoneal space after gynecologic surgery to reduce postoperative adhesions. Citing a higher infection rate and questionable clinical benefit in a placebo-controlled trial, an advisory committee voted against approving Intergel in 2000 [55]. The FDA shortly followed with a non-approvable decision, prompting Lifecore to request a MDDRP meeting. In 2001, when presented with a modified indication and additional data from an animal safety study, the panel voted in favor of approval, which the FDA subsequently granted (it is not obligated to follow MDDRP recommendations). Less than two years later, Lifecore removed Intergel from the market after reports of repeat operations for pain, foreign body reactions, and tissue adherence [56].

## Discussion

Advances in medical device technologies have translated into significant improvements in the health of patients. Yet cracks in the device review system may threaten to undermine this progress. Our analysis has identified eight specific potential weaknesses in the premarket review process (Table 1). Although each is considered separately, these weaknesses often interact with one another. Moreover, three overarching issues provide the context in which these weaknesses take place.

First, the 1997 amendments direct the agency to consider the “least burdensome” means of showing effectiveness for devices [57],[58], giving the industry recourse to challenge many requests it regards as onerous. For example, ReGen invoked this language when the FDA considered the unfavorable findings of its RCT, asserting that because the agency was “required to consider the least burdensome information necessary to demonstrate substantial equivalence,” an analysis relying upon the RCT was “at odds with the Act” [59]. However, even this assertion was incorrect, as the relevant language for 510(k)s is only applicable to situations involving different technological characteristics (see Issue 3), which did not apply to ReGen.

Second, user fees paid by the industry for device review bind the FDA to specific review time goals [60]. These fees—valued at roughly $49 million in FY2008 or approximately one-sixth of the device review budget [60]—shift the agency from being merely a regulator to being financially dependent upon the very industry it is charged with regulating.

Third, the FDA appears to have permitted scientific approaches that fall short of rigorous. Approaches drawn from the exemplars put forth in this article include post hoc subgroup analyses [40], historical [35] and non-concurrent controls [22], and unnecessary unblinding [22],[35]. In addition, the FDA report on the review of the MCS shows inappropriate involvement in the scientific review process from Congresspeople and the then-FDA commissioner [27].

Addressing the eight issues will require, in our opinion, remedial actions in three dimensions—legislative, regulatory, and agency practice—and certain problems will be susceptible to more than one approach.

On the legislative front, US Congress should raise the standard for approval of devices intended to treat diseases to equal that required for drugs: “substantial evidence” rather than “reasonable assurance” of effectiveness. Such devices should be subject to the same regulatory scrutiny as drugs, such as more than one well-controlled trial. While it is rarely used, the de novo process is a legislative loophole that requires tightening. The safety and effectiveness of devices cleared through this pathway have been demonstrated in neither clinical studies nor by reference to a predicate device. Finally, devices with different technological characteristics are, by definition, dissimilar and evaluating such devices using the 510(k) route is therefore inappropriate. Congress should also repeal this statutory provision, steering such devices toward the PMA route.

With respect to regulation, we believe the agency should define criteria for “same intended use” in a more limited manner. Doing so could prevent certain novel devices from proceeding through the 510(k) pathway. Furthermore, the agency should adhere to existing laws and regulations. For example, it should expeditiously complete classification of class III preamendments devices, as well as all unclassified devices missed in the initial classification effort. Shortly thereafter, the FDA should call for PMA applications for any device retained in class III.

The existing legislative and regulatory framework for premarket review inevitably leaves many crucial decisions open to FDA interpretation. As the issues reviewed in this article demonstrate, this discretion has been applied in an expansive manner favorable to Industry. CDRH could address these weaknesses through shifts in its discretionary practices, such as insisting on higher scientific standards, tightening the interpretation of “same intended use,” and insisting on more-rigorous review procedures in those cases where the optimal review pathway is a matter of judgment rather than law.

Opponents of such changes might argue that equal treatment of drugs and devices intended to treat diseases would place an undue burden upon typically smaller device companies, potentially keeping important products from entering the market. However, as is true for drugs, larger companies often acquire startups that produce promising devices. Moreover, the FDA's mission is to protect the public health, and allowing questionably effective products onto the market seems inconsistent with that mission.

Some might also argue that whereas drugs are static entities once approved (indeed, any change in the chemical nature of the product requires a new application), devices tend to advance incrementally. But an existing abbreviated mechanism – PMA supplements – permits design changes to be made without unduly burdening device manufacturers each time a modification is made.

Methodological issues unique to device studies—primarily unblinding and sample size—are often raised as defenses of the current regulatory regime. The broad acceptance of this argument creates a milieu in which unnecessarily lax scientific standards continue to be accepted [61],[62]. Rather than treating these as justifications for reduced rigor, they should be regarded as factors to be considered in interpreting study results.

A series of problems with the FDA's premarket regulation of devices at times appears to permit potentially unsafe or ineffective devices to reach the market. Each must be remedied with a mix of legislative, regulatory, and discretionary approaches unique to that problem. Most importantly, CDRH should place its decisions on a secure evidence base.

## Abbreviations

CDRH: Center for Devices and Radiologic Health
ECT: electroconvulsive therapy
FDA: Food and Drug Administration
FDAMA: Food and Drug Administration Modernization Act
FDCA: Food, Drug and Cosmetic Act
MCS: Menaflex Collagen Scaffold
MDA: Medical Device Amendments
MDDRP: Medical Device Dispute Resolution Panel
NDA: new drug application
PMA: premarket approval
RCT: randomized controlled trial
SMDA: Safe Medical Devices Act
TMS: transcranial magnetic stimulation
VNS: vagus nerve stimulator
